# Transcriptional responses of *Metarhizium pingshaense* blastospores after UV-B irradiation

**DOI:** 10.3389/fmicb.2024.1507931

**Published:** 2024-12-05

**Authors:** Amanda Rocha da Costa Corval, Lucas Amoroso Lopes de Carvalho, Emily Mesquita, Jéssica Fiorotti, Thaís Almeida Corrêa, Victória Silvestre Bório, Adriani da Silva Carneiro, Daniel Guariz Pinheiro, Irene da Silva Coelho, Huarrisson Azevedo Santos, Everton Kort Kamp Fernandes, Isabele da Costa Angelo, Vânia R. E. P. Bittencourt, Patrícia Silva Golo

**Affiliations:** ^1^Postgraduate Program in Veterinary Sciences, Veterinary Institute, Federal Rural University of Rio de Janeiro, Rio de Janeiro, RJ, Brazil; ^2^Laboratory of Bioinformatics, Department of Agricultural, Livestock and Environmental Biotechnology, School of Agricultural and Veterinary Sciences, São Paulo State University (UNESP), Jaboticabal, SP, Brazil; ^3^Graduate Program in Agricultural and Livestock Microbiology, School of Agricultural and Veterinary Sciences, São Paulo State University (UNESP), Jaboticabal, SP, Brazil; ^4^Department of Biochemistry and Immunology, Ribeirão Preto School of Medicine, University of São Paulo, Ribeirão Preto, SP, Brazil; ^5^Department of Veterinary Microbiology and Immunology, Veterinary Institute, Federal Rural University of Rio de Janeiro, Rio de Janeiro, RJ, Brazil; ^6^Department of Epidemiology and Public Health, Veterinary Institute, Federal Rural University of Rio de Janeiro, Rio de Janeiro, RJ, Brazil; ^7^Institute of Tropical Pathology and Public Health, Federal University of Goiás, Goiânia, GO, Brazil; ^8^Department of Animal Parasitology, Veterinary Institute, Federal Rural University of Rio de Janeiro, Rio de Janeiro, RJ, Brazil

**Keywords:** entomopathogenic fungi, insect control, RNA-seq, tolerance to UV-B, bioinputs

## Abstract

*Metarhizium* is widely known for its role as an arthropod biocontrol agent and plant bioinoculant. By using mass-production industrial methods, it is possible to produce large amounts of fungal single-celled propagules (including blastospores) to be applied in the field. However, in the environment, the solar ultraviolet components (particularly UV-B) can harm the fungus, negatively impacting its pathogenicity toward the arthropod pest. The present study is the first to use comparative genome-wide transcriptome analyses to unveil changes in gene expression between *Metarhizium pingshaense* blastospores exposed or not to UV-B. Relative blastospores culturability was calculated 72 h after UV-B exposure and exhibited 100% culturability. In total, 6.57% (*n* = 728) out of 11,076 predicted genes in *M. pingshaense* were differentially expressed after UV-B exposure: 320 genes (44%; 320/728) were upregulated and 408 (56%; 408/720) were downregulated in the UV-B exposed blastospores. Results unveiled differentially expressed gene sets related to fungal virulence, production of secondary metabolites, and DNA repair associated with UV damage; genes related to virulence factors were downregulated, and genes associated with nucleotide excision repair were upregulated. These findings illustrate critical aspects of *Metarhizium* blastospores strategies to overcome UV-B damage and survive solar radiation exposures in insulated fields.

## 1 Introduction

Entomopathogenic fungi are known for their capacity to infect and frequently lethally affect arthropod hosts upon contact with their cuticle. These fungi are safe for vertebrates and can be isolated from infected arthropods and soil samples (Correa et al., 2022). They are among the most widely used organisms for biological control due to their ease of mass production and application, similar to synthetic pesticides (Mascarin et al., 2019). Besides their action against arthropods, these fungi are also considered plant symbionts, acting as biofertilizers and bioinoculants of crops (Mesquita et al., 2023).

*Metarhizium* can produce various propagules, including aerial or submerged conidia, blastospores, mycelium, and microsclerotia. The production of these different propagules is driven by factors such as nutrient availability, temperature, humidity, and the physical properties of the growth medium. Solid substrates favor the production of aerial conidia, while liquid cultures under specific conditions can induce the formation of submerged conidia, blastospores, or microsclerotia (Marciano et al., 2021; Garcia-Riaño et al., 2024).

Blastospores are thin-walled yeast-like unicellular hydrophilic spores that can be easily suspended in water, in contrast to aerial conidia. Blastospores are produced under *in vitro* conditions in submerged liquid fermentation and differ from hyphal bodies, propagules formed inside the arthropod’s body during the initial infection stage. Blastospore mass production is cheaper and faster than the production of aerial conidia (Alkhaibari et al., 2017); additionally, this propagule generally germinates faster than conidia and has multiple routes of infection (particularly in mosquitos), which is believed to be a determinant for their higher or similar virulence compared to aerial conidia (Alkhaibari et al., 2016; Gomes et al., 2023). All these characteristics stimulate the research and prospection of this propagule in a world where the bioproducts market is under constant growth. Although traditional solid substrate fermentation is still the most common method for producing and commercializing important entomopathogenic fungi, submerged liquid fermentation technology is believed to supplement or even replace it soon (Mascarin et al., 2024).

The success of biological pest control using fungi depends on several factors, including the mechanisms by which the entomopathogenic fungus behaves in the face of various stresses. One of these environmental stresses is the incidence of ultraviolet (UV) radiation on the Earth’s surface, impacting the survival of microorganisms in the field. UV radiation is conventionally divided into three distinct regions: UV-A (315–400 nm), UV-B (280–315 nm), and UV-C (100–280 nm) (Sliney, 2007). Although UV-C has a shorter wavelength and higher energy, it is almost entirely filtered by the ozone layer and thus poses no direct threat under field conditions. Only UV-A and UV-B penetrate the Earth’s surface; while UV-A is more abundant than UV-B, the former has lower energy and causes less damage to DNA and cellular structures. Despite the amount of UV-B represents less than 1% of the radiation that reaches the Earth (Jenkins, 2017), this DNA absorption can cause irreversible damage to entomopathogenic fungi, inducing dimerization between adjacent pyrimidine bases, which can be either the most frequent cyclobutane pyrimidine dimers or the least-frequent pyrimidine (6–4) pyrimidone photoproducts (Brancini et al., 2022). Formation of base dimers can lead to fungus death and reduced germination speed and virulence, negatively impacting the effectiveness of fungi as bioinsecticides (Brancini et al., 2022). Additionally, literature has shown that exposure to UV-B can activate DNA repair mechanisms in widely used entomopathogenic fungi, such as *Beauveria bassiana* and *Metarhizium* spp (Brancini et al., 2022; Feng, 2024). Genetic studies with *Metarhizium* spp. also have been conducted to unveil the role of specific genes for the fungus’ UV-B tolerance (Zhu et al., 2018; Gao et al., 2020; Song et al., 2022).

New molecular tools and analysis characterized the entomopathogenic fungus *Metarhizium anisopliae* (Hypocreales: Clavicipitaceae) and sister species. For instance, *Metarhizium pingshaense* is part of *Metarhizium* clade 1, formed by the species *M. anisopliae*, *Metarhizium brunneum*, *Metarhizium guizhouense*, *Metarhizium majus*, *M. pingshaense*, and *Metarhizium robertsii* (Schneider et al., 2011). A recent study revealed the expression pattern of *M. pingshaense* photolyase (an enzyme related to photoreactivation repair), different hours after blastospores UV-B exposure, giving insights on what repair mechanisms may be linked to the germination of this fungus after UV exposure (Lima et al., 2024). Despite this, there are subjects in this study area yet to be addressed. Some examples include changes in the expression of genes and metabolic signatures of *Metarhizium* blastospores after UV-B exposure, mainly related to this fungus’ virulence, production of secondary metabolites, and DNA recovery after UV-B damage. Accordingly, transcriptome analysis becomes essential to unveiling *Metarhizium*’s molecular responses to UV-B stress. Through high-throughput RNA sequencing, it is possible to identify differentially expressed genes contributing to understanding fields of inquiry. This approach may help clarify how *Metarhizium* adapts to UV-B-induced stress, offering valuable information for selecting isolates with greater environmental resistance and, therefore, greater efficacy as biocontrol agents.

Transcriptomic studies on *Metarhizium* are less abundant than those on more commonly studied organisms like model fungi (i.e., Saccharomyces cerevisiae) or human pathogens. Despite this, interest in entomopathogenic fungi is growing, especially with increasing awareness of the need for sustainable agricultural practices. As technologies advance and the significance of biocontrol agents is more widely recognized, more transcriptomic studies on *Metarhizium* have been emerging in the literature, such as Gao et al. (2011), Brancini et al. (2019), Gotti et al. (2023), Iwanicki et al. (2023), and Liu et al. (2023). Despite this, none of them address the consequences of post-UV-B stress. The present study hypothesizes that UV-B exposure alters gene expression in *M. pingshaense* blastospores (leading to changes in the expression of genes involved in virulence, secondary metabolite production, and DNA repair mechanisms) and these changes may, in turn, affect the fungus’ ability to infect arthropod hosts. Accordingly, the present study aimed to investigate the transcriptome profile and differential gene expression in blastospores of *M. pingshaense* exposed to UV-B radiation through high-throughput RNA sequencing, focusing on aspects related to the fungal infection process in arthropod hosts.

## 2 Material and methods

### 2.1 *Metarhizium pingshaense* LCM S10

*Metarhizium pingshaense* LCM S10 was used in the present study. The entomopathogenic fungus was isolated from a soil sample, collected in Rio de Janeiro state, Brazil (Lima et al., 2024). The isolate was cultivated in potato dextrose agar, supplemented with 0.1 g/l yeast extract (PDAY), and incubated under controlled conditions [25 ± 1°C and relative humidity (RH) ≥ 80% for 14 days in the dark]. The Petri dishes were then stored at 4°C for further use. As the present study accessed Brazilian genetic heritage, the research was registered at the Brazilian National System for the Management of Genetic Heritage and Associated Traditional Knowledge (Sisgen) under the code AA47CB6.

### 2.2 Blastospores production

Blastospores production followed the methodology described by Lima et al. (2024) with adjustments. Fourteen-day-old cultures of *M. pingshaense* LCM S10 conidia were scraped from PDAY plates and suspended in 0.01% (v/v) polyoxyethylene sorbitan monooleate (Tween 80^®^) sterile distilled water solution. For blastospores production, three ml of 1 × 10^8^ conidia ml^–1^ were inoculated into 50 ml of potato dextrose broth (Kasvi^®^) supplemented with 0.1 g/l yeast extract. The flasks were capped with hydrophobic cotton and placed on an orbital shaker (TE-424; Tecnal^®^) at 200 rpm for 72 h at 25 ± 1°C. After 72 h, the medium was filtered in a funnel with sterile gauze to remove the mycelium produced during the culture, and blastospores suspensions were transferred to 50 ml centrifuge tubes. The medium containing blastospores underwent two cycles of centrifugation at 3,410 × *g* for 5 min (Rotina 380R Hettich^®^). After the first cycle, the supernatant was discarded, and the pellet was suspended in 10 ml 0.01% (v/v) Tween 80^®^ sterile distilled water solution, followed by vortex homogenization and centrifugation. After the second centrifugation, the supernatant was discarded, and the pellet was suspended in 5 ml 0.01% (v/v) Tween 80^®^ aqueous solution, followed by vortex homogenization. Suspensions were adjusted to 1.0 × 10^6^ ml^–1^ and transferred to empty Petri dishes.

### 2.3 Exposure of *Metarhizium pingshaense* blastospores to UV-B radiation

The experiments were carried out according to Corval et al. (2021) and Lima et al. (2024). Two fluorescent lamps TL 20W/12 RS (Philippines, Eindhoven, Netherlands) provided UV-B radiation. Ten ml of blastospores aqueous suspension (1.0 × 10^6^ ml^–1^) were placed in a 23-ml Petri plate (90 × 15 mm, Kasvi). Plates with blastospores were exposed to UV-B radiation for approximately one hour (total fluence = 4.0 kJm^–2^). During irradiation, plates were covered with a 0.13-mm thick cellulose diacetate film (JCS Industries), which blocks UV-C radiation (below 280 nm) and the UV-B short-wavelength (280–290 nm) but permits the passage of most UV-B (290–320 nm) and the minimal UV-A (320–400 nm) radiation emitted by the lamps (Supplementary Figure 1). Control plates were also placed in the chamber but covered with aluminum foil to block all UV radiation. The temperature inside the chamber was controlled (25 ± 1°C). The DNA damage action spectrum (pyrimidine dimerization) normalized for the unit at 300 nm was used to calculate the UV irradiance (Quaite et al., 1992; Braga et al., 2001). The radiation spectrum was measured using the Ocean Optics USB 2000 Spectroradiometer (Dunedin, FL).

Part of the propagules (9.9 ml) were used for RNA extraction, and the other part was adjusted to 1.0 × 10^4^ blastospores/ml. A 20-μl aliquot of blastospores aqueous suspension was inoculated onto Petri plates with PDAY and spread by using a glass rod. Plates were incubated with a photoperiod of 12/12 h at 27 ± 1°C. Colony forming units (CFUs) were quantified 72 h after UV-B exposure. The relative culturability was calculated as previously described by Braga et al. (2001). The experiments were performed three times with different batches of blastospores.

The relative culturability data were submitted to the Shapiro–Wilk test to check the normality. The CFUs were analyzed using analysis of variance (ANOVA) followed by the Skott–Knott test considering *P* < 0.05 and using GraphPad Prism, v.8.4.0, Inc. (GraphPad Software).

### 2.4 RNA extraction and quantification

RNA extraction from blastospores was performed immediately after exposure to UV-B, using and according to the PureLink™ RNA Mini Kit protocol. For each condition (UV-B exposed and control), three biological replicates, originating from different batches of blastospores, were used. Following extraction, samples were treated with DNAse I (Thermo Fisher Scientific, Wilmington, DE, USA). An electrophoresis run was performed to verify the samples’ extraction quality and any DNA contamination. The RNA was quantified by fluorometry using the Qubit RNA BR assay (Thermo Scientific^®^, Waltham, Massachusetts, EUA). The samples were analyzed at GenOne Soluções em Biotecnologia, Rio de Janeiro, RJ, Brazil.

### 2.5 RNA-seq

Total RNA samples were fragmented and purified using oligo-linked poly-T magnetic beads, and cDNA was synthesized using random hexamer primers followed by second-strand cDNA synthesis. Illumina DNA library preparation and sequencing were outsourced to GenOne: Soluções em Biotecnologia, Rio de Janeiro, Brazil. The library was constructed after final repair, A-tailing, adapter ligation, size selection, amplification, and purification. The library was checked with Qubit^®^ (Thermo Fisher Scientific, USA), q-PCR for quantification, and a bioanalyzer for size distribution detection. Illumina paired-end reads (2 × 150 bp) were produced using the NovaSeq6000 platform.

### 2.6 RNA-seq data analysis

#### 2.6.1 Data processing

Processing started by assessing the quality of the data sequenced using the “FastQC” software (v.0.11.9) (Andrews, 2010). The “Atropos” program (v.1.1.24) (Didion et al., 2017) was used to remove residual sequencing adapters. A two-step processing was implemented with “Atropos,” the first one based on the “insert” mode, which checks for adapters in the excess overlapping, and the second in the “adapter” mode, a standard search for alignment between the adapter sequence and the target sequence, in a semi-global way. To remove low-quality sequences and bases, the “PRINSEQ-lite” program (v.0.20.4; Schmieder and Edwards, 2011) was used, in which bases whose window consisted of 3 consecutive bases (“-trim_qual_window 3”), with a Phred (Q) quality value lower than 30 (“-trim_qual_right 30”), as well as complete sequences whose average quality was lower than Q 30 (“-min_qual_mean 30”) or less than 50 bases (“-min_len 50”). Sequences whose respective pairs were lost during the quality control steps (“singletons”) were reserved and used in later steps.

#### 2.6.2 Determination of expressed genes

Sequences were aligned against the genome of *M. robertsii* ARSEF 23 (GCF_000187425.2) with the “STAR” program (v.2.7.1a; Dobin and Gingeras, 2015). The program was configured to consider introns with sizes between 30 (“—alignIntronMin 30”) and 300 (“-alignIntronMax 300”) base pairs (values obtained based on the annotation file of genetic characteristics of the reference genome) and a maximum of 20 multiple alignments (“-outFilterMultimapNmax 20”). Additionally, we conducted a supplementary alignment against the recently released *M. pingshaense* M-1000 genome (GCA_041379975.1). Alignment metrics for both genomes are provided in Supplementary Table 3. The aligned libraries of the “paired-end” (PE) and “singletons” sequences were merged and submitted to the “Cuffquant” program (v.2.2.1; Trapnell et al., 2012) to quantify the gene expression of each sample based on the number of mappings. To avoid library size variation bias, expression values were normalized to FPKM (“Fragments per kilobase of exon per million mapped reads”) with the “Cuffnorm” program (v.2.2.1), which also estimated absolute counts to be used in differential expression analysis. The raw data has been deposited at the NCBI Sequence Read Archive database under the ID PRJNA1127190.^1^

#### 2.6.3 Identification of differentially expressed genes

The differentially expressed genes (“DEGs”) were identified with the R package “DESeq2” (v. 1.34.0; Love et al., 2014) which applies the Wald’s test for statistical analysis. To correct for multiple testing, we used the false discovery rate (FDR) method, setting a q-value (adjusted *p*-value) threshold of 0.05 to define significant DEGs. DEGs with low expression differences were filtered, establishing a minimum Log2 Fold-Change value of ± 1. DEGs were visualized using volcano plots, which were constructed with log2 fold-changes and -log10 FDR-adjusted *p*-values using the ggplot2 (v. 3.3.6; Wickham, 2016) and ggrepel (v.0.9.4; Slowikowski, 2024) packages.

#### 2.6.4 Annotation and analysis of functional enrichment

In addition to the genetic annotation information present in the GFF file of the reference genome (*M. robertsii* ARSEF 23, GCF_000187425.2), an additional annotation was performed against the EggNOG ortholog database (v. 5.0.2; Huerta-Cepas et al., 2016), using the “eggnog-mapper” tool (v. 2.1.8; Huerta-Cepas et al., 2016). This annotation allowed us to assign functional information to the predicted genes, categorizing them into clusters of orthologous genes (COGs) to provide an overview of their functional roles. Additionally, associations of expressed genes with GO terms (gene ontology) were obtained, which were then subjected to enrichment analysis using the “GOATOOLS” suite of tools (v. 1.2.3; Klopfenstein et al., 2018). The enrichment analysis was performed separately for up-regulated and down-regulated DEGs, using Fisher’s exact test to assess GO term enrichment. A significance threshold of *P* < 0.05 was applied in both cases to identify overrepresented GO terms.

#### 2.6.5 Principal component analysis and graphical representations

To reduce the dimensionality and define the principal components regarding the variation of the differential expression profiles of the samples, a principal component analysis (PCA) was used, implemented by the “prcomp” function of the R “stas” package (v. 4.1.2; R Core Team, 2020). The “heatmap” type graph was generated with the “pheatmap” R package (v. 1.0.12; Kolde, 2019) and the others, with the “ggplot2” R package (v. 3.3.6; Wickham, 2016).

### 2.7 Validation of RNA-seq data

Seven differentially expressed transcripts in the large-scale sequencing were randomly selected to have their expression pattern confirmed by a real-time PCR assay. The primers were designed using Primer Express 3.0 software (Thermo Fisher Scientific, Wilmington, DE, USA) and the transcripts obtained from the transcriptome itself (Supplementary Table 1). Each primer-pair’s efficiency was assessed through standard curves constructed with six-fold serial dilutions of *M. pingshaense* cDNA following the instructions described in the Minimum Information for Publication of Quantitative Real-Time (MiQE) guidelines (Vandesompele et al., 2002). Translation elongation factor 1α (TEF) was the reference gene (Golo et al., 2015).

For the qPCR assay, RNA was converted into cDNA by the High-Capacity cDNA Synthesis Kit (Thermo Fisher Scientific, Wilmington, DE, USA). The qPCR assay was performed using the Step One Plus Real-Time PCR System (Applied Biosystems, Thermo Fisher). Each sample (control or UV-treated) was tested in triplicate using customized primers (Thermo Fisher Scientific, Wilmington, DE, USA) at 0.4 μM sense and antisense, 1× Power SYBER Green kit (Thermo Fisher Scientific, Wilmington, DE, USA), and 60 ng/μl cDNA in a total volume of 12 μl. The cycling conditions were as follows: an initial cycle of denaturation at 95°C for 10 min, followed by 40 cycles of 95°C for 15 s and 60°C for 1 min. Fluorescence readings were taken at 60°C after each cycle, and a dissociation curve (60–95°C) was performed. A negative standard control was prepared with molecular-grade water. The Spearman corrkelation test was applied to verify the correlation between the mRNA levels from RNA-seq and the qPCR technique.

## 3 Results

### 3.1 UV-B tolerance of *Metarhizium pingshaense* blastospores

The effect of the UV-B exposure on the culturability of *M. pingshaense* LCM S10 blastospores was accessed by analyzing CFUs on exposed and control plates. The relative culturability of blastospores in the three repetitions was 100%.

### 3.2 Overview of RNA-seq data

The RNA-seq quality was considered satisfactory by the FastQ quality check, and most of the sequences were between 30 and 40 on the Phred scale. On average, 12,164,853 reads were obtained per control library and 11,962,780 per UV-B exposed. After the pre-processing steps that included adapter removal and quality control, a reduction of approximately 1.38% of the original counts was observed (Supplementary Table 2).

**FIGURE 1:**
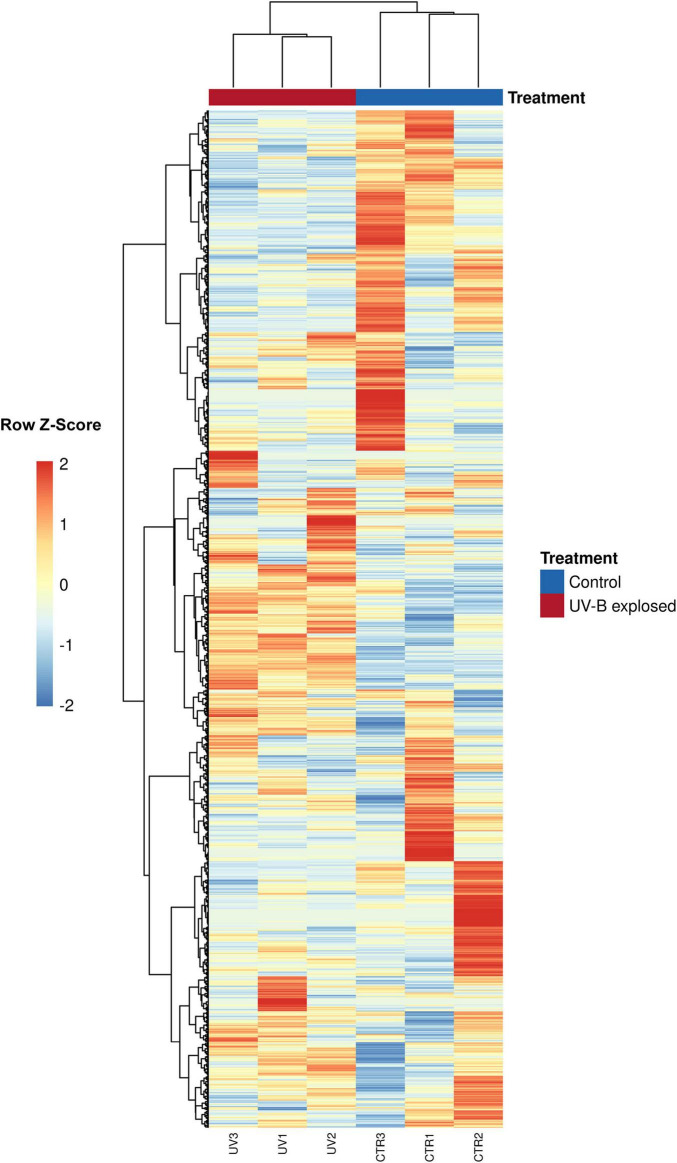
Heatmap visualization of RNA-seq data from *Metarhizium pingshaense* blastospores exposed or not exposed to UV-B. For each sample in the RNA-seq dataset, the figure includes a heatmap demonstrating gene expression. Each row of the heatmap represents a gene, each column represents a sample, and each cell displays normalized gene expression values. Blue color represents low expressed genes, and red color represents highly expressed genes (FDR adjusted *p* < 0.05; Log2FC > 1 or < –1).

Comparisons between unexposed propagules (CTR) and exposed propagules (UV-B) were made to determine the main genes related to *M. pingshaense* blastospores’ response to UV-B exposure. The sequence assembly resulted in 72,382,900 putative transcripts distributed among six samples (three controls and three UVB-exposed). The sequences were aligned against both the *M. robertsii* ARSEF 23 genome (GCF_000187425.2) and the recently released *M. pingshaense* M-1000 genome (GCA_041379975.1). While both genomes provided high alignment rates, the alignment metrics with *M. robertsii* ARSEF 23 were slightly superior (Supplementary Table 3), and its gene annotation allowed the reliable identification of genes for our study. Although the alignment to the reference genome (*M. robertsii* ARSEF 23) resulted in an alignment of over 99% of good quality sequences, the mean unique alignment rates of CTR and UV-B reads to the reference genome were 65.89 and 60.31%, respectively (Supplementary Table 3). The reference genome had 11,688 predicted genes, of which 11,076 (94.76%) were detected as expressed by the sequencing (Supplementary Table 4). Figure 1 exhibits the heatmap summarizing all expressed genes.

**FIGURE 2:**
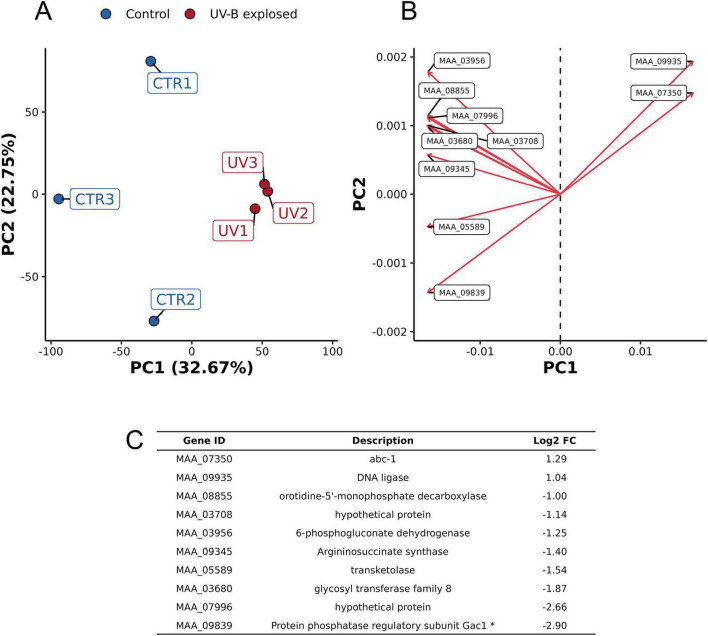
Principal component analysis of the transcriptome for comparison of *Metarhizium pingshaense* blastospores exposed or not to UV-B. **(A)** Score plot of PC1 and PC2. Control samples are black dots, and UV-B exposed samples are orange dots. **(B)** Loading plot exhibiting the ten genes with the highest loadings in PC1 (mainly related to the separation of treatments). **(C)** Descriptions and differences in expression of the ten genes. Positive numbers indicate upregulated genes, and negative numbers indicate down-regulated genes after UV-B exposure. The asterisk (*) indicates a description based on orthologs obtained from annotation with EggNOG.

The conclusion drawn from the principal component analysis (PCA), considering all genes, suggests that the three replicates from the UV-exposed groups can be easily grouped considering PC1 and PC2 (Figure 2A). In contrast, there was more variation between the control samples, mainly related to PC2 (22.75%) (Figure 2A). As expected, the control and the UV-treated groups exhibited considerable variation (Figure 2A), mainly related to PC1 (32.67%). The accumulated variation of the two first PCs’ eigenvalues was 55.42%. The top 10 genes with the highest loading on PC1 are represented in the loading plot (Figure 2B). These genes are described in Figure 2C. Positive numbers indicate upregulated genes, and negative numbers indicate down-regulated genes after UV-B exposure.

**FIGURE 3:**
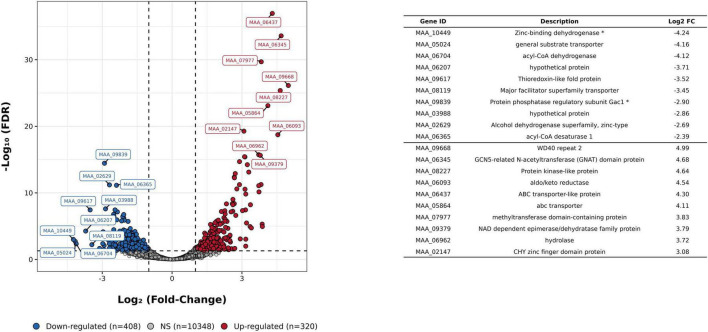
Comparative transcriptome analysis of *Metarhizium pingshaense* blastospores exposed to UV-B. The volcano plot shows the differential gene expression in blastospores exposed to UV-B compared to that in unexposed blastospores (CTR). The data points above the significance threshold (FDR adjusted *p* < 0.05; Log2FC > 1 or < –1) are marked in blue (down-regulated in UV-B) and red (upregulated in UV-B), and others are marked in gray (not significant).

Differential expression analysis showed that out of these 11,076 genes, 728 (6.57%) were differentially expressed between CTR and UV-B (FDR adjusted *p* < 0.05; Log_2_FC > 1 or < −1). Of these, 320 genes (2.88% of the expressed genes) were upregulated, and 408 (3.68% of the expressed genes) were downregulated in the UV-B compared to the CTR (Figure 3). Information on expression values and functional annotation of differentially expressed genes are in Supplementary Table 5.

### 3.3 Validation of RNA-seq data

Seven genes were selected for the validation of RNA-seq results. The Spearman coefficient (ρ) revealed a strong and significant correlation between RNA-seq and qPCR data (ρ = 0.78/1) (*p*-value < 0.0001).

### 3.4 Functional analysis of transcriptome profiles

**FIGURE 4:**
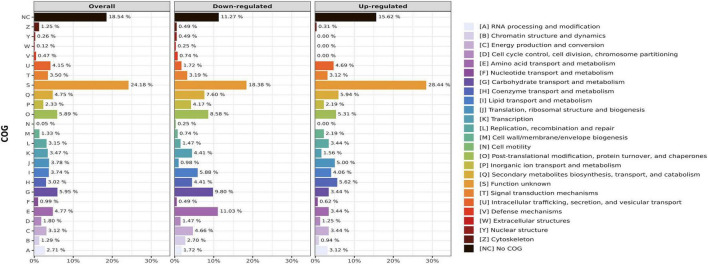
Cluster of orthologous groups of proteins (COG) classification of *Metarhizium pingshaense* blastospores DEGs after exposure to UV-B (total fluence 4 kJ m-2).

To investigate the overall patterns of gene function amongst DEGs after the UV-B exposure in *M. pingshaense* blastospores, a gene set enrichment analysis of the main biological processes, cell components, and molecular function was performed using the clusters of orthologous groups (COG) and gene ontology (GO). The functional classification of DEGs reveals that 88.72% (362/408) of the down-regulated and 84.37% (270/320) of the upregulated genes could be assigned to at least one of the COG categories. However, 18.38% (75/408) of the down-regulated genes and 28.44% (91/320) of the upregulated genes had an unknown function. The categories with the highest number of down-regulated genes were: “[E] Amino acid transport and metabolism” (11.03%), “[G] Carbohydrate transport and metabolism” (9.80%), and “[O] Post-translational modification, protein turnover, and chaperones” (8.58%). For those upregulated, the main categories were: “[Q] Secondary metabolites biosynthesis, transport, and catabolism” (5.94%), “[H] Coenzyme transport and metabolism” (5.62%), and “[O] Post-translational modification, protein turnover, and chaperones” (5.31%) (Figure 4).

**FIGURE 5:**
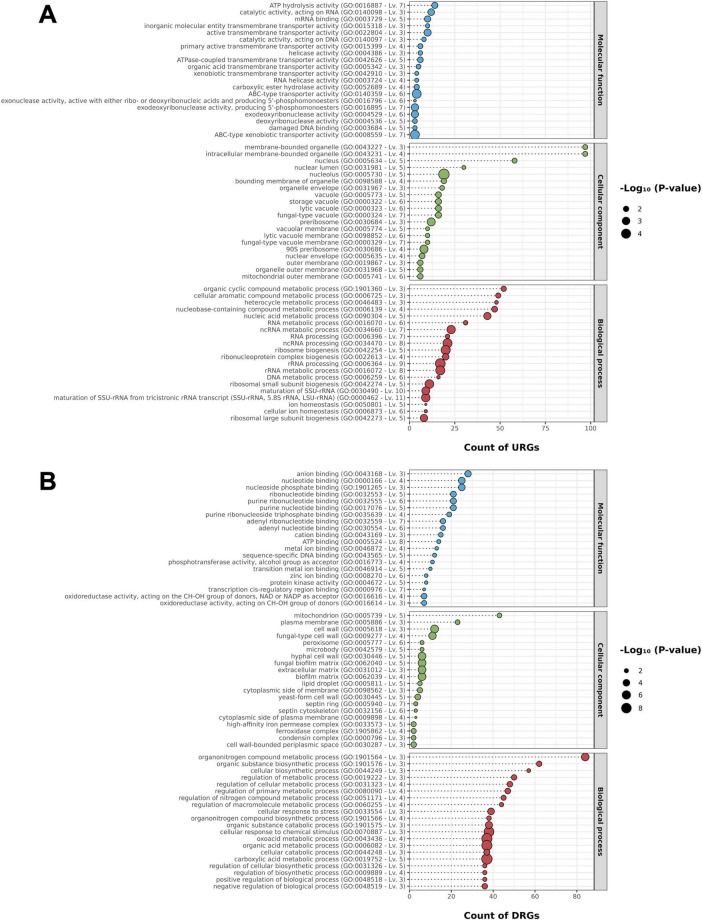
Enrichment analysis of gene ontology (GO) Terms of **(A)** up- and **(B)** down-regulated genes of *Metarhizium pingshaense* blastospores after UV-B exposure. The percentages of differentially expressed genes in the ten most expressive terms of each functional category.

Based on all DEGs, GO term enrichment analysis resulted in a total of 459 significantly enriched terms (*P* ≤ 0.05) (Supplementary Table 6). The DEGs were evaluated separately to ascertain the enrichment of specific terms in those up or downregulated. As a result, a total of 218 GO terms enriched for upregulated genes and 787 GO terms for down-regulated genes were obtained (Supplementary Table 6). The most relevant GO terms for each part are observed in Figure 5.

To complement these gene set enrichment analyses, we further inspected the functional annotations of the DEGs. This process allowed us to identify genes of interest related to fungal virulence, the production of secondary metabolites, and DNA repair mechanisms associated with UV damage. The following paragraphs provide a detailed description of the main DEGs involved in these biological processes.

#### 3.4.1 Expression of *Metarhizium* virulence factors

By studying how UV exposure changes the expression of *Metarhizium* virulence factors, it is possible to gain insights into the dynamics of gene regulation under environmental stresses. Accordingly, the present study investigated whether there were expression differences in pathogenicity-related genes between UV-B exposed blastospores and unexposed propagules. Proteins such as the insect cuticle binding adhesin MAD1, are important for regional localization of adhesive propagule surfaces. Here, the mad1 gene (MAA_03775) was downregulated (log_2_ fold change: −1.1, FDR-adjusted *P* = 0.002) in UV-B exposed blastospores compared to unexposed propagules (Supplementary Table 5).

Subtilisin-like proteases of insect-pathogenic fungi are a large family of extracellular cuticle-degrading enzymes often considered virulence factors since they are involved with fungal invasion into insect hemocoel through classic cuticle infection. One out of the 15 subtilisin-like proteases genes expressed in *M. pingshaense* blastospores was upregulated in UV-B exposed blastospores (log_2_ fold change: 2.3, FDR-adjusted *P* < 0.0001, gene: MAA_10425) (Supplementary Table 5). No subtilisin-like serine protease genes were differentially expressed in UV-B exposed propagules. Another group of proteases known to be involved in the pathogenicity of entomopathogenic fungi are trypsin-like serine proteases (Dubovenko et al., 2010). One out of ten trypsin-like proteases genes expressed in *M. pingshaense* blastospores was down-regulated in UV-B exposed blastospores, the Peptidase cysteine/serine trypsin-like protein (log_2_ fold change: −1.3; FDR-adjusted *P* = 0.004, gene: MAA_10345) (Supplementary Table 5).

Once the entomopathogenic fungus invasion into the host is successful, the next challenge is fighting against the host’s immune defense mechanisms. One of the fungus’ strategies is hiding to prevent the host’s phagocytosis, which is mediated mainly by hemocytes in the hemolymph. *Metarhizium* blastospores can express the collagen-like protein MCL1 (Wang and St Leger, 2006), which is a protein that provides an antiadhesive protective coat that masks beta-glucan components of the cell wall preventing detection by hemocytes. According to our results, one of the two identified *mcl1*genes expressed in *M. pingshaense* blastospores was downregulated in UV-B exposed propagules, the Collagen-like protein MCL1 gene (log_2_ fold change: −1.3; FDR-adjusted *P* = 0.0008, gene: MAA_01665) (Supplementary Table 5).

#### 3.4.2 *Metarhizium* secondary metabolites and detoxification

One of the best-known types of secondary compounds produced by *Metarhizium* is destruxins (Golo et al., 2014). Here, the destruxin synthetase DtxS1 gene was downregulated in blastospores after the UV-B exposure (MAA_10043, log_2_ fold change: −1.4; FDR-adjusted *P* = 0.003) (Supplementary Table 5). Nevertheless, among the genes in the GO category *secondary metabolite biosynthetic processes* (GO:0044550), there were genes upregulated (MAA_06572, log_2_ fold change: 2.2; FDR-adjusted *P* = 0.0003; MAA_06566, log_2_ fold change: 1.9; FDR-adjusted *P* = 0.0005; MAA_01653, log_2_ fold change: 2.0; FDR-adjusted *P* = 0.002) and genes down-regulated (MAA_02182, log_2_ fold change: −1.1; FDR-adjusted *P* = 0.003; MAA_09697, log_2_ fold change: −1.3; FDR-adjusted *P* = 0.0005).

The biosynthesis of fungal metabolites is based on three large classes of core biosynthetic genes: polyketide synthase, non-ribosomal peptide synthetases, and terpene biosynthetic genes (Keller and Hohn, 1997). In the present study, none of the genes encoding non-ribosomal peptide synthetases or terpene biosynthetic genes were differentially expressed. Out of the 22 genes expressing polyketide synthase, four were downregulated in UV-B exposed blastospores (MAA_01701, log_2_ fold change: −2.0; FDR-adjusted *P* = 0.002; MAA_08518, log_2_ fold change: −1.8; FDR-adjusted *P* < 0.0001; MAA_10033 log_2_ fold change: −2.4; FDR-adjusted *P* = 0.0003; MAA_10220, log_2_ fold change: −1.2; FDR-adjusted *P* = 0.002) (Supplementary Table 5).

In fungi, Cytochrome P450 (CYP) proteins play pivotal roles in facilitating metabolic versatility, enabling adaptation to specific ecological niches, including the host’s inside (Huarte-Bonnet et al., 2018). Out of the 125 CYP genes, five were differentially expressed in *M. pingshaense* blastospores exposed to UV-B. Three genes were upregulated (MAA_03841, log_2_ fold change: 1.8; FDR-adjusted *P* < 0.0001; MAA_08354, log_2_ fold change: 1.6; FDR-adjusted *P* = 0.001; MAA_08378, log_2_ fold change: 2.1; FDR-adjusted *P* = 0.0008) and two genes were downregulated (MAA_08689, log_2_ fold change: −3.9; FDR-adjusted *P* = 0.0002; MAA_06628, log_2_ fold change: −1.6; FDR-adjusted *P* = 0.0008) (Supplementary Table 5).

#### 3.4.3 UV damage repair

Once the fungal DNA absorbs UV-B, damage can be induced in the DNA chemical bases, resulting in base dimers such as cyclobutane pyrimidine dimmers or the less-frequent pyrimidine (6–4) pyrimidone photoproducts. The fungus can use dark repair (i.e., general nucleotide excision repair and specific nucleotide excision by UV-endonucleases) (Sinha and Hader, 2002) or photoreactivation repair to repair damaged DNA bases (Brancini et al., 2018). In the present study, photolyase (the enzyme activated during photoreactivation repair) (gene MAA_05216) was not differentially expressed between blastospores exposed or not to UV-B (Supplementary Table 4). Similarly, the gene that encodes the white-collar protein WC1 (MAA_04453), that together with the WC2 form a light-sensitive factor that regulates the expression of photolyase (Peng et al., 2021; Tong and Feng, 2022), was not differentially expressed (Supplementary Table 4). On the other hand, three genes out of the 22 encoding DNA repair proteins were upregulated in *M. pingshaense* blastospores exposed to UV-B (gene MAA_02816, DNA repair protein Rad26, log_2_ fold change: 1.5; FDR-adjusted *P* = 0.004; gene MAA_03026, HhH-GPD superfamily base excision DNA repair protein, log_2_ fold change: 1.5; FDR-adjusted *P* = 0.0001; and MAA_06158, DNA repair protein RAD16, log_2_ fold change: 1.2; FDR-adjusted *P* = 0.0007) (Supplementary Table 5).

## 4 Discussion

The infection process of *Metarhizium* in arthropod hosts is usually divided into four steps: (a) propagule adhesion on the hosts’ cuticle; (b) penetration into the cuticle, toward the inside of the host; (c) mass-propagation and secretion of toxins in the insect body; and (d) sporulation and dispersion after egressing from the host. Nevertheless, before penetration into the arthropod, the fungi should overcome abiotic stresses, such as UV, which can interfere with their physiology even when they do not kill them (Rangel et al., 2015). This is the first study that focuses on *M. pingshaense* response to UV-B exposure, unveiling valuable information that can be further explored in a more in-depth understanding of the effect of UV-B exposure on the regulation of genes in *Metarhizium* blastospores.

Their low-cost mass production drove the use of blastospores in the present study and evidence that these propagules can actively penetrate the arthropods’ cuticle (Bernardo et al., 2018). Here, the relative culturability of *M. pingshaense* LCM S10 blastospores exposed to UV-B was 100%, which means all propagules were able to grow into fungal colonies even after the UV-B exposure; however, it is worth mentioning that the culturability analysis was performed 72 h after the exposure, so these propagules had three days to recover from the UV stress. Additionally, the propagule’s growth does not indicate there was no impact on the fungus’ ability to infect the arthropod. The expression of genes related to the infection process of the fungus into the arthropod was highlighted to unveil the implications of UV-B exposure for *M. pingshaense* blastospores virulence and capacity to bypass the arthropod’s immune system.

A previous study reported that the disruption of the adhesion protein MAD1 in *M. anisopliae* delayed germination, suppressed blastospore formation, and greatly reduced virulence against caterpillars (Wang and St Leger, 2007) since it impacts the first step in the infection process. Accordingly, it is critical to unveil if UV-B negatively impacts *mad1* (MAA_03775), and other genes’ expression, as these genes are implicated in affecting fungal virulence. In the present study, the *mad1* gene and the polyketide synthase genes MAA_01701, MAA_08518, MAA_10033, and MAA_10220 were downregulated in blastospores after UV exposure (Supplementary Table 5). Polyketide synthase is an enzyme important for conidial germination, and thus virulence, in entomopathogenic fungi, including *Metarhizium* (Huang et al., 2022) and *Beauveria* (Meng et al., 2021). The reduced expression of these gene sets suggests UV-B can highly impair *Metarhizium* infection capacity, reducing mortality rates which may negatively impact the fungus’ pathogenicity in the field, for example. Despite this, even right after the UV-B challenge, some *Metarhizium* blastospores will probably be able to adhere and germinate to the arthropod’s surface. The literature on the effect of UV on entomopathogenic fungi reported that UV exposure can also contribute to the emergence of mutants with higher virulence than the unexposed propagules (Zhao et al., 2016; Sun et al., 2023). These authors screened surviving colonies after UV challenging, and studied these mutants. Accordingly, while UV exposure appears to impair critical genes linked to entomopathogenic fungal adhesion and germination, it may also create selective pressures that could, paradoxically, drive the emergence of more resilient and potentially virulent fungal variants.

The fungus will need proteases to penetrate the host’s cuticle following adhesion. Entomopathogenic fungi use various enzymes to penetrate the host cuticle (Arnesen et al., 2018). Among them are subtilisin-like serine proteases produced to degrade chitin-associated proteins in the arthropod’s cuticle. According to our results, only one subtilisin-like protease was upregulated (MAA_10425) and no subtilisin-like serine protease genes were differentially expressed after the UV-B exposure, suggesting that this type of radiation does not impact the expression of subtilisin-like serine proteases. As for the genes encoding trypsin-like proteases, another critical group of enzymes that facilitates entomopathogenic fungal penetration into the arthropod host, one (MAA_10345) out of the ten genes expressed was downregulated in exposed blastospores. Together, these results suggested there is no standard response in *Metarhizium* blastospores caused by UV radiation on the expression of genes related to cuticle degradation.

As soon as the fungus invades the arthropod host, it is necessary to avoid its immune response. The *Metarhizium* collagen-like protein MCL1 is crucial for evading host immunity and for the fungus adaptation to defenses in new hosts (Wang and St Leger, 2006). Here, as observed for the *mad1* gene, one *mcl1* gene was downregulated in UV-B exposed propagules. Again, it is possible to suggest that the stress management orchestrated by *M. pingshaense* blastospores in response to UV-B may negatively impact the fungus pathogenicity. Following the infection process, after expressing the MCL1 onto the surface of *Metarhizium* cells to avoid recognition and encapsulation by arthropods immune cells, the fungus can express the MOS1 receptor protein to adapt to the high osmotic pressure of the insect hemolymph (Wang et al., 2008). Although the Osmosensor Mos1 gene (MAA_01571) was expressed in *M. pingshaense* blastospores (Supplementary Table 4), it was not differentially expressed after UV exposure, suggesting this mechanism of adaptation is not affected by UV-B.

Once the fungus enters the arthropod host and evades its primary immune response, it eventually secretes many secondary metabolites to interfere with, inhibit, or counter the host immune response to kill the arthropod. Many studies have been published on the role of destruxins in the infection process of entomopathogenic fungi in arthropods highlighting their role as virulence factors against insects (Sree and Padmaja, 2008; Yin et al., 2021; Wang et al., 2023). In the present study, the destruxin synthetase DtxS1 gene (MAA_10043) was downregulated in blastospores after the UV-B treatment, emphasizing one more negative impact of UV-B on *M. pingshaense* blastospores exposed to UV-B. However, among the genes in the GO category secondary metabolite biosynthetic processes (GO:0044550), there were genes up and downregulated.

CYP genes are another set of genes that should be examined in the context of fungal infection. CYP enzymes may exhibit fatty acid hydroxylase activity, contributing to virulence and growth of the entomopathogenic fungus on insect cuticles (Zhang et al., 2012). Fungi can encode several CYP enzymes; as example, the CYP52 clan can has up to 10 gene families (CYP52, CYP538, CYP539, CYP584, CYP585, CYP655, CYP656, CYP5087, CYP5113, and CYP5203). CYP52 clan is implicated in the first hydroxylation step in alkane-assimilation processes. In contrast, genes belonging to the clan CYP53 (a single-family clan) have been linked with the oxidation of aromatic hydrocarbons (Huarte-Bonnet et al., 2018). No genes from the CYP52 clan were differentially expressed in the present study. However a gene belonging to the family CYP53 (Cytochrome P450 CYP539B4; MAA_06628) was downregulated along with CYP55 (Cytochrome P450 CYP55A20; MAA_08689). On the other hand, three genes expressing the CYPs CYP5058, CYP6003, and CYP68 were upregulated (Supplementary Table 5). According to Gao et al. (2011), CYP53 gene-family members from *M. anisopliae* and *M. acridum* were upregulated during insect-cuticle infection. In the present study, downregulation of MAA_06628 (that encodes CYP539B4) in UV-B exposed blastopores compared to unexposed propagules suggests a negative impact on the fungal virulence caused by UV-B exposure. Although other CYPs were upregulated, the meaning of this modulation cannot be completely unveiled since not all entomopathogenic fungi CYP families and subfamilies have their role completely disclosed.

The sun’s ultraviolet components (particularly UV-B) can be harmful to non-living matter and living organisms, including fungi (Corval et al., 2021). This damage often results in the formation of base dimers such as cyclobutane pyrimidine dimers, which are more common, or the less common pyrimidine (6–4) pyrimidone photoproducts, both generated via covalent linkages of adjacent bases (Sinha and Hader, 2002). The fungus can repair this damage through general or specific nucleotide excision by UV-endonucleases or photolyase activated by photoreactivation light (320–500 nm) to fix the damaged DNA bases (Brancini et al., 2021; Feng, 2024). As expected, the DNA-repair photolyase gene was not differentially expressed when exposed to UV-B, and unexposed blastospores were compared (Lima et al., 2024). As previously published by our group, an increase in the *M. pingshaense* photolyase gene expression occurred only between 24 and 36 h after UV-B exposure of blastospores (with a boost in expression 36 h after exposure), suggesting the blastospores recovery observed until 24 h after the stress might not be coordinated by this enzyme (Lima et al., 2024). In the present study, the blastospores’ RNA was extracted right after the UV-B exposure. Accordingly, the fungus had no time to start the recovery process using photolyase. However, there was up-regulation of three genes related to nucleotide excision repair (MAA_02816, MAA_03026, and MAA_06158) (Supplementary Table 5). The upregulated genes encode proteins RAD16, RAD 26, and HhH-GPD superfamily. Although the role of not all RAD proteins is unveiled in *Metarhizium*, recent literature reported that RAD26 contributes significantly to UV-B resistance and function as nucleus-specific proteins essential for the recovery of damage in *M. robertsii* caused by solar UV exposure (Peng et al., 2024).

The transcriptional responses of *M. pingshaense* to UV-B exposure provide valuable insights into how these fungi may survive under natural field conditions with solar exposure. Our analysis suggested that while UV exposure may reduce the fungus’s immediate pathogenicity, it may also create selective pressures that drive the emergence of more resilient fungal variants. Understanding these molecular adaptations is critical for improving the long-term survival and effectiveness of *Metarhizium* as a biocontrol agent in diverse environmental conditions. Additionally, given the negative impact of UV-B on key virulence-related genes, it is crucial to investigate strategies to enhance the UV-B resistance of *Metarhizium* such as formulation or genetic modification.

## 5 Conclusion

The present study contributed to unveiling the gene expression profile of *M. pingshaense* blastospores in response to UV-B exposure. Based on the analysis of gene expression related to virulence factors in blastospores after UV-B exposure, and considering the survival of *Metarhizium* blastospores following the irradiation, we assumed that by adjusting virulence-related genes, the fungus could improve its repair processes and stress responses to better survive the exposure to UV-B stress. This adaptation, however, comes at a significant cost, as it reduces the fungus’ ability to infect and kill target arthropods.

## Data Availability

The datasets presented in this study can be found in online repositories. The names of the repository/repositories and accession number(s) can be found in this article/Supplementary material.

## Footnotes

1
https://www.ncbi.nlm.nih.gov/sra/?term=prjna1127190

## Supplementary material

Supplementary Figure 1 Irradiance spectrum of the cellulose diacetate-filtered lamps of the UV-B irradiation chamber. The lamps provided 967.01 mW/m2 of the UV-B irradiation at the exposure-shelf level in the chamber, based on Quaite-weighted irradiance.

Supplementary Table 1 Gene ID, name, primer sequences, and PCR efficiency of the genes used in the qPCR to validate the transcriptome.

Supplementary Table 2 Summary of *Metarhizium pingshaense* LCM S10 RNA-seq read filtering and mapping.

Supplementary Table 3 Alignment metrics for *Metarhizium robertsii* ARSEF 23 (GCF_000187425.2) and *Metarhizium pingshaense* M-1000 (GCA_041379975.1) genomes.

Supplementary Table 4 Complete dataset of the gene differential expression analysis and its statistics for all genes mapped against the *Metarhizium robertsii* ARSEF 23 genome.

Supplementary Table 5 Dataset with significantly differentially expressed genes in *Metarhizium pingshaense* LCM S10 blastospores after UV-B exposure.

Supplementary Table 6 Enrichment analysis of gene ontology (GO) terms of up- and down-regulated genes of *Metarhizium pingshaense* blastospores after UV-B exposure.
