# Supplementary figures and images for: Transcriptional responses of Metarhizium pingshaense blastospores after UV-B irradiation
Source: Front Microbiol. 2024 Dec 5;15:1507931. doi: 10.3389/fmicb.2024.1507931 (PMC11656200; doi:10.3389/fmicb.2024.1507931)

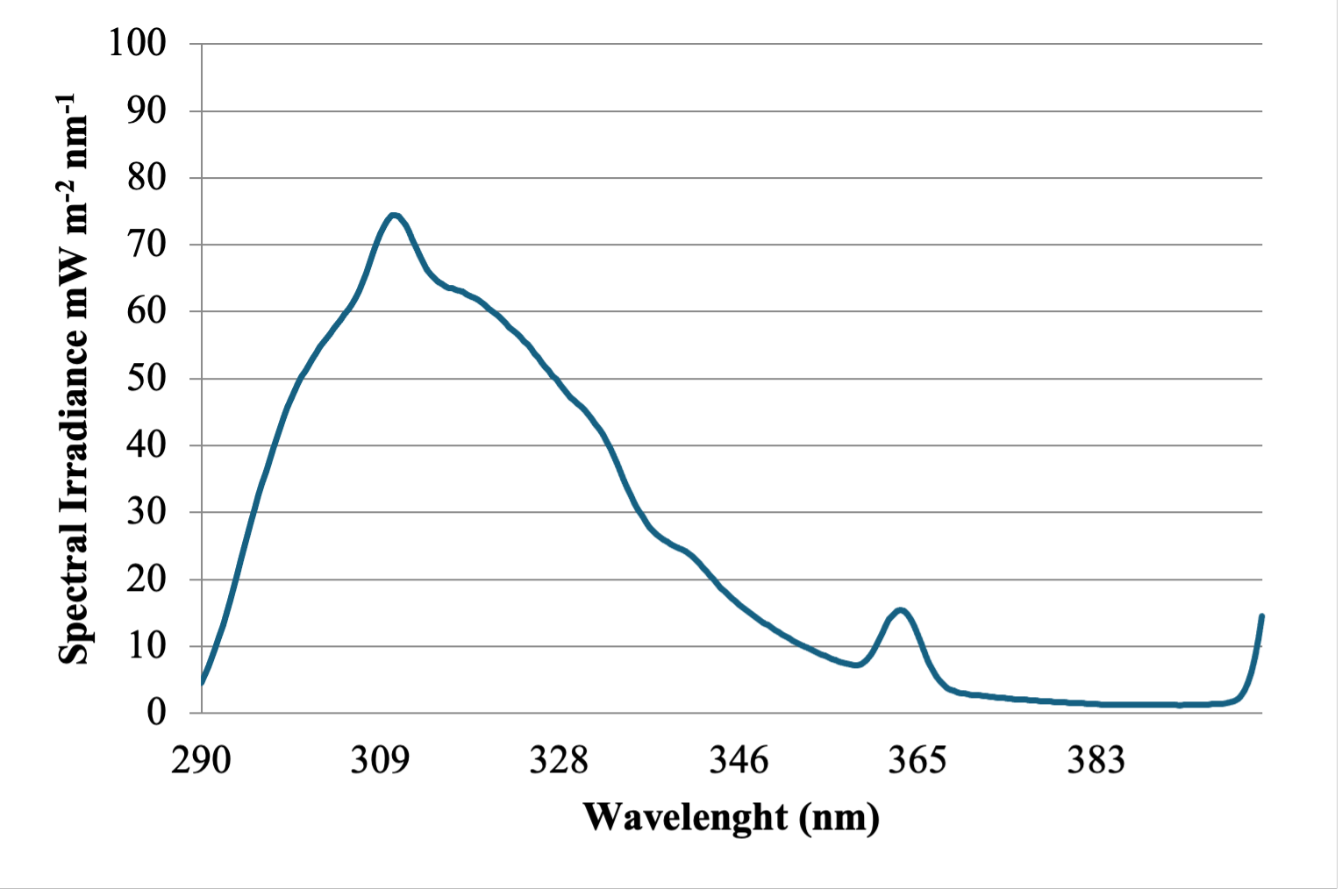

Supplement: Supplementary Figure 1 — Irradiance spectrum of the cellulose diacetate-filtered lamps of the UV-B irradiation chamber. The lamps provided 967.01 mW/m2 of the UV-B irradiation at the exposure-shelf level in the chamber, based on Quaite-weighted irradiance. [file Image_1.tiff]
