# Supplementary material for: Transcriptional responses of Metarhizium pingshaense blastospores after UV-B irradiation
Source: Front Microbiol. 2024 Dec 5;15:1507931. doi: 10.3389/fmicb.2024.1507931 (PMC11656200; doi:10.3389/fmicb.2024.1507931)
Supplement: Supplementary Table 1 — Gene ID, name, primer sequences, and PCR efficiency of the genes used in the qPCR to validate the transcriptome. [file Table_1.pdf]

Supplementary Table S1. Gene ID, name, primer sequences, and PCR efficiency of the genes used in the qPCR to validate the transcriptome.

Gene ID (GenBank)	Gene name	Forward (5′–3′) and reverse (5′–3′) primers	PCR Efficiency (%)
MAA_05864	*Metarhizium robertsii* ARSEF 23abc transporter	F CACAGACCCCGATGAGATGTT<br>R CTTCAGTTCAGGCGTAACGAGTT	96.6
MAA_06300	*M. robertsii* 23MFS transporter	F TCGCCAACTTCCTACTCAAGAAA<br>R CAGCAACCCCACAAGATCATG	95.1
MAA_06704	*M. robertsii* 23 acyl-CoAdehydrogenase	F AGTGGCTAGGTCCGCTGCTA<br>R GCAACTTGAGGTTCCGTCATG	97.3
MAA_09668	*M. robertsii* ARSEF 23WD40 repeat 2	F CATCACGAAGCTCGAAATCG<br>R CGTCCATTTGGGCAAACG	98.5
MAA_01920	*M. robertsii* ARSEF 23 RNA helicase, DEAD-box type, Q motivos protein	F CCAGTTGGACGTGCCGTACT<br>R GCCAAAATCTTCACCCACTGA	94.8
MAA_10425	*M. robertsii* ARSEF 23subtilisin-like protease [Metarhizium robertsii 23]	F GATTGGGTCCAACAGCTTGAG<br>R TGCGTTTCGTGTCTAGTCTTGAG	97.7
MAA_05024	*M. robertsii* ARSEF 23 general substrate transporter	F GAAGATGCTGAAGCAAATGGT<br>R TTGGCTCTCAAGCTTTTCATTAGA	95.8
